# Gallstone Ileus: An Unusual Cause of Intestinal Obstruction

**DOI:** 10.7759/cureus.7284

**Published:** 2020-03-15

**Authors:** Tia Morosin, Marie Shella B De Robles, Soni Putnis

**Affiliations:** 1 Surgery, The Wollongong Hospital, Wollongong, AUS; 2 Surgery, Philippine General Hospital, Manila, PHL

**Keywords:** gallstone ileus, cholelithiasis, cholecystoduodenal fistula, laparotomy, enterotomy, intestinal obstruction

## Abstract

Gallstone ileus is an uncommon complication of gallstones and a rare cause of intestinal obstruction. Typically as a result of the formation of cholecystoduodenal fistula, surgical removal of the gallstone is the mainstay of treatment in order to relieve the intestinal obstruction. A 34-year-old male with no history of cholelithiasis presented with features of a small bowel obstruction. CT scan of the abdomen demonstrated pneumobilia, a cholecystoduodenal fistula and small bowel obstruction, features suspicious for a gallstone ileus. The patient underwent a laparotomy and removal of two gallstones via an enterotomy. He was discharged home after an uneventful post-operative period. Gallstone ileus is an uncommon cause of mechanical bowel obstruction with often delayed presentation and non-specific symptoms. A high level of suspicion is required in at-risk groups, and in patients presenting with a bowel obstruction and known gallstone disease.

## Introduction

Gallstone ileus is a rare cause of intestinal obstruction, occurring in less than 5% of patients who present with a mechanical small bowel obstruction [1]. Gallstone ileus is an unusual complication of cholelithiasis. It is caused by the impaction of a gallstone in the small bowel, usually after passing through a biliary-enteric fistula typically formed between the gallbladder and duodenum [2]. Female and older patients are disproportionality affected, and a high index of suspicion should be needed when patients present with a bowel obstruction and known history of gallstones [1,3]. Here we present a case of a gallstone ileus in a patient with no preceding history who underwent a laparotomy with enterolithotomy. 

## Case presentation

**Figure 1:**
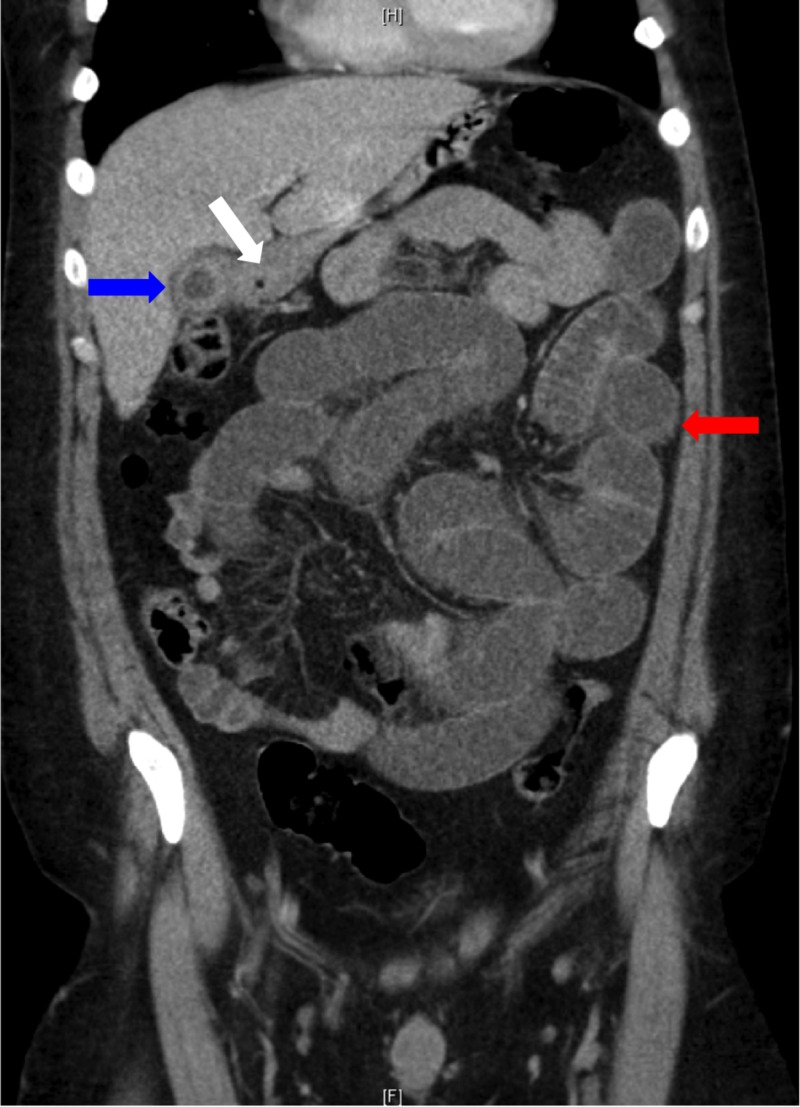
Dilation of intrahepatic ducts (white arrow), pneumobilia (white arrow), contracted gallbladder with thickened wall (blue arrow), dilated loops of small bowel (red arrow)

**Figure 2:**
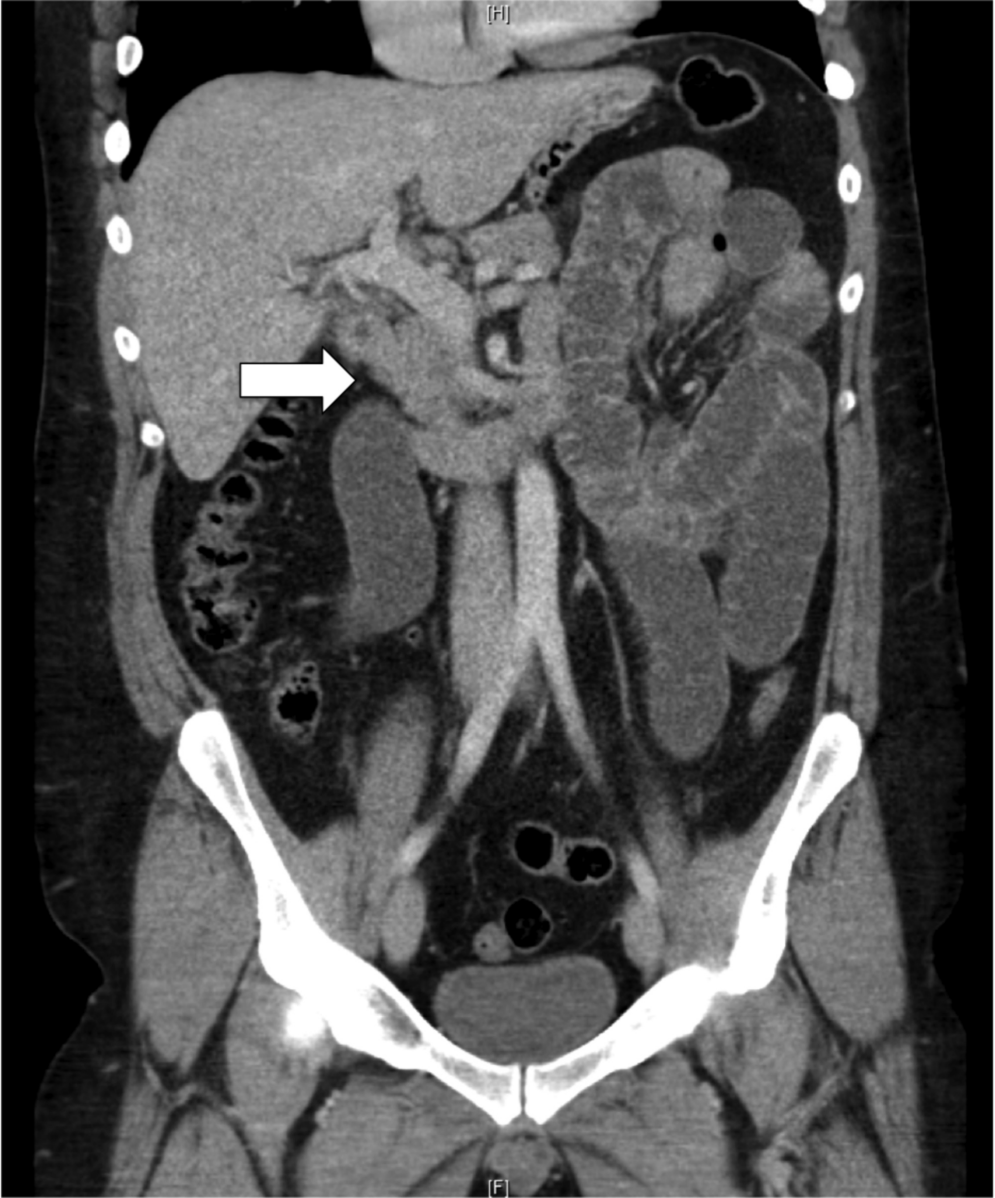
Cholecystoduodenal fistula formation (white arrow)

A 34-year-old male presented with a one-day history of colicky epigastric pain and vomiting. He also reported two days of constipation and not passing flatus. An otherwise healthy male, he had no previous medical history, denied any previous biliary symptoms, no history of cholelithiasis and no previous abdominal surgery. He was haemodynamically stable and afebrile on presentation. Examination revealed a soft abdomen with moderate distension and epigastric tenderness; however, no rebound tenderness or guarding was noted. Routine blood tests were unremarkable. CT of the abdomen demonstrated a small bowel obstruction with the point of obstruction in mid abdomen and a cholecystoduodenal fistula suspicious for gallstone ileus; however, no radio-opaque stone was seen (Figures 1, 2).

**Figure 3:**
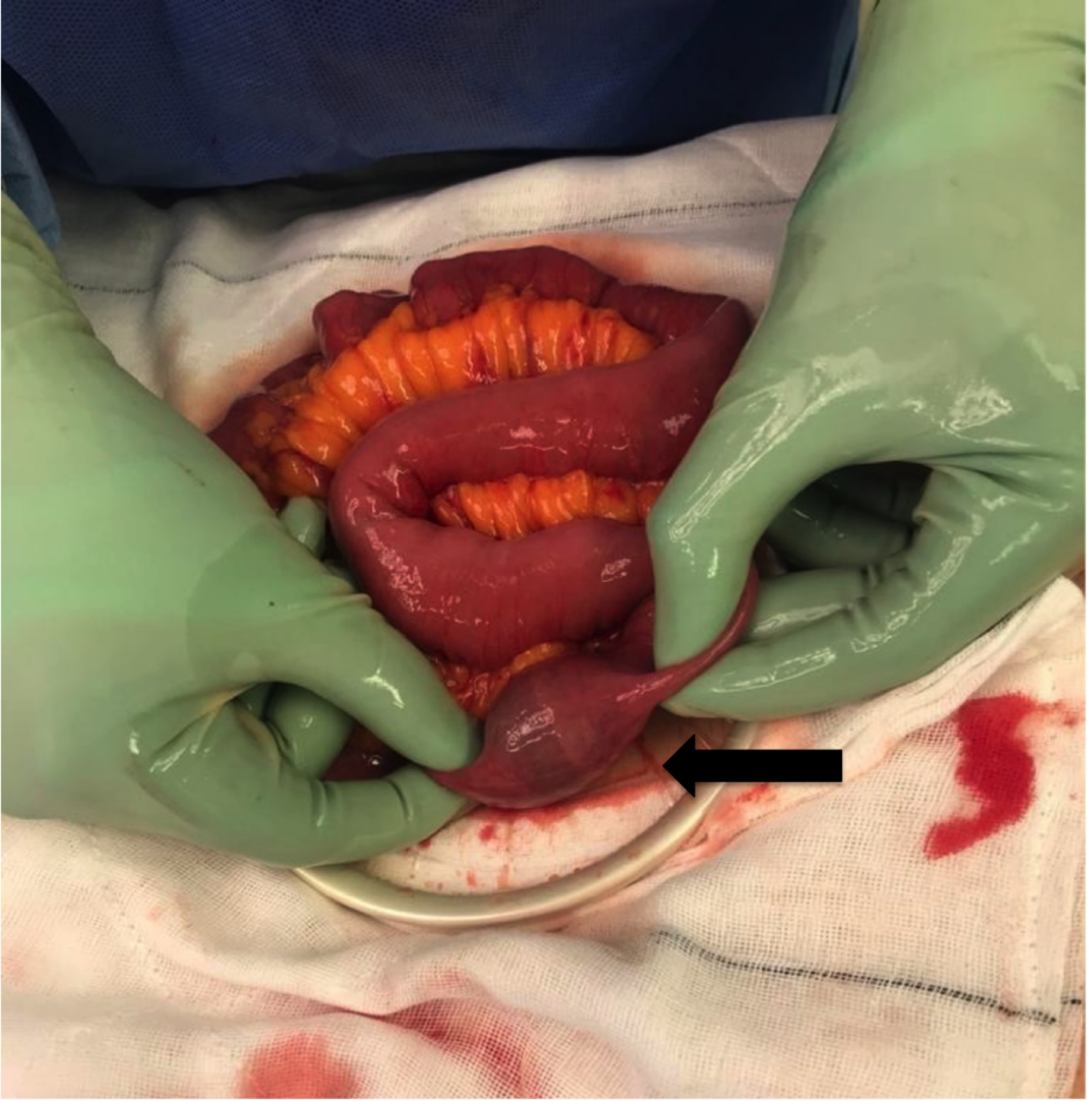
Impacted gallstone (black arrow)

**Figure 4:**
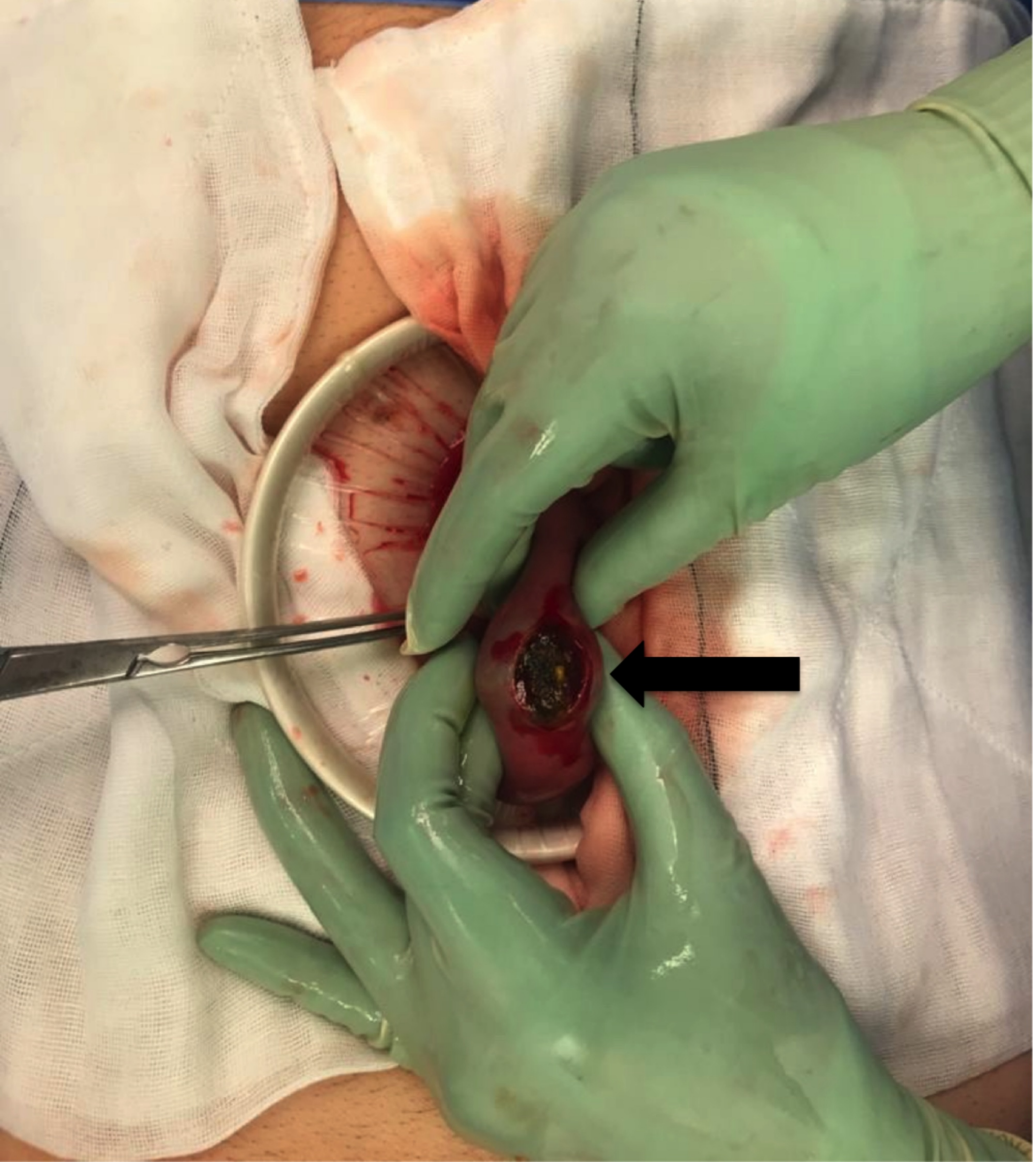
Small bowel enterotomy for removal of the impacted gallstones (black arrow)

He was resuscitated with intravenous fluids and had a nasogastric tube inserted for decompression. The patient underwent a laparotomy. Intraoperative findings noted small bowel obstruction with the transition point at 50 cm from the ileocaecal valve caused by two large gallstones obstructing the lumen (Figure 3). A longitudinal 1 cm enterotomy was made proximal to the distal gallstone (Figure 4). Both stones were removed (2 and 3 cm), and the enterotomy was closed transversely. His post-operative period was uneventful, and the patient was discharged home day 3 post-operatively.

## Discussion

Although labelled as an ileus, impaction of gallstones in the small intestine is a true mechanical obstruction. It is rare in nature, accounting for less than 5% of mechanical obstructions, and is associated with significant morbidity and mortality (overall 18%) [1]. It is also an uncommon complication of cholelithiasis, occurring in 0.3%-0.5% of patients with gallstones, most commonly the elderly and female population [1,3]. While not apparent in the case presented, the majority of cases are preceded by acute cholecystitis resulting in the formation of a biliary-enteric fistula. Pericholecystic inflammation results in the formation of adhesions between the gallbladder and the gastrointestinal tract, usually the duodenum, due to proximity. The pressure effect of the gallstone results in erosion of the stone through the gallbladder wall into the intestine forming the fistula tract as presently demonstrated [2,4]. The formation of a biliary-enteric fistula allows the entry of gallstones into the gastrointestinal system and complicates 0.3%-1.5% of cases of cholelithiasis [1]. Alternatively, the gallstone may pass through the common bile duct into the duodenum through the ampulla [2,5]. Most stones lodge in the ileum (60.5%), the narrowest segment of the bowel; reduced peristalsis in this area has also been suggested to be a contributing factor. Stones may also lodge in the jejunum (16.1%), stomach (14.2%) and less commonly the duodenum (3.5%) [1,2,4,6]. Most reported cases of obstruction demonstrate a gallstone larger than 2 cm in diameter, with smaller stones potentially able to pass through the intestine to the rectum [2-4,7,8]. As discovered in the present case, multiple gallstones may be present in up to 40% of cases [3]. The nature of the obstruction often results in non-specific and intermittent signs and symptoms. Clinical examination is consistent with a bowel obstruction, dehydration, nausea, vomiting, abdominal distension, pain and high-pitched bowel sounds, which are most commonly present [2,4,9]. Patients may delay in presenting in part due to the “tumbling phenomenon”. It describes the intermittent nature of symptoms secondary to temporary gallstone impaction followed by symptom relief when the stone dislodges, travels distally and impacts again [1,2,4,10]. While the reported patient had no history of cholelithiasis, up to 30% of cases present with concurrent acute cholecystitis at the time of obstruction [2]. Patients may also present tachycardic, hypotensive and febrile, signs suggestive of sepsis from either cholecystitis or peritonitis due to impaction of the gallstone causing pressure to the bowel wall, necrosis and perforation [2]. Biochemical markers may be unremarkable or non-specific and may include leukocytosis and deranged electrolytes [3,10]. As such, a high level of suspicion in patients with a history of gallstones presenting with a bowel obstruction is required. Rigler’s triad describes classical features seen on imaging suggestive of gallstone ileus: (1) intestinal obstruction, (2) pneumobilia, (3) gallstone within the intestinal lumen and, more recently, a change in position of the gallstone on serial imaging [11]. Apart from traditionally x-ray findings, the features are also apparent on CT abdomen scan, which, with sensitivity and specificity of 93% and 100%, respectively, has become the gold standard [12]. A CT scan may also help visualise the viability of the bowel, detect features of cholecystitis (although not diagnostic) and, as in the current case, identify the presence of a biliary-enteric fistula, thereby helping to guide the management of the patient. The aim of treatment is to relieve the obstruction, which centres on removal of the gallstone. While there is consensus regarding the need for enterolithotomy, there is controversy in the literature as to whether a cholecystectomy and fistula repair should be performed concurrently. Of the two procedures, there is a known increased risk of morbidity and mortality associated with a single-stage procedure; however, there is also the risk of further complications from gallstones while awaiting a cholecystectomy and repair of fistula in a two-staged procedure [2-6,9]. Intraoperatively, our patient was noted to have a chronically inflamed and contracted gallbladder with a large fistula tract. Consequently, we elected for a staged procedure with a laparotomy and enterolithotomy during the initial presentation. 

## Conclusions

Gallstone ileus is an uncommon cause of mechanical bowel obstruction with often-delayed presentation and non-specific symptoms. As such a high level of suspicion is required in at-risk groups and in patients presenting with a bowel obstruction and known gallstone disease. 
